# Recent HIV Infection among men who have sex with men and transgender women in Tijuana

**DOI:** 10.11606/s1518-8787.2020054002179

**Published:** 2020-08-24

**Authors:** Britt Skaathun, Heather A. Pines, Thomas L Patterson, Shirley J Semple, Jonathan Pekar, Alicia Harvey-Vera, Gudelia Rangel, Sanjay R. Mehta

**Affiliations:** I University of California Department of Medicine Division of Infectious Diseases and Global Public Health San DiegoLa Jolla USA University of California, San Diego. Department of Medicine. Division of Infectious Diseases and Global Public Health. San Diego, La Jolla, USA; II University of California Department of Psychiatry San DiegoLa Jolla USA University of California, San Diego. Department of Psychiatry. San Diego, La Jolla, USA; III University of California Bioinformatics and Systems Biology Program San DiegoLa Jolla USA University of California, San Diego. Bioinformatics and Systems Biology Program. San Diego, La Jolla, USA; IV Universidad Xochicalco TijuanaBaja California Mexico Universidad Xochicalco. Tijuana, Baja California, Mexico; V United States-Mexico Border Health Commission TijuanaBaja California Mexico United States-Mexico Border Health Commission. Tijuana, Baja California, Mexico; VI El Colegio de la Frontera Norte TijuanaBaja California Mexico El Colegio de la Frontera Norte. Tijuana, Baja California, Mexico; VII University of California Department of Pathology San DiegoLa Jolla, USA University of California, San Diego. Department of Pathology. San Diego, La Jolla, USA; VIII San Diego Veterans Affairs Medical Center Department of Medicine San Diego USA San Diego Veterans Affairs Medical Center. Department of Medicine. San Diego, USA

**Keywords:** HIV Infections, epidemiology, Sexual and Gender Minorities, Transgender Persons, Disease Transmission, Infectious

## Abstract

**OBJECTIVE:**

To characterize recent HIV infections among newly diagnosed men who have sex with men and transgender women in Tijuana.

**METHODS:**

Limiting Antigen (LAg)-Avidity testing was performed to detect recent HIV infection within a cohort of newly-diagnosed men who have sex with men and transgender women in Tijuana. Logistic regression was used to determine characteristics associated with recent infection. A partial transmission network was inferred using HIV-1 *pol* sequences. Tamura-Nei 93 genetic distances were measured between all pairs of sequences, and the network was constructed by inferring putative transmission links (genetic distances ≤ 1.5%). We assessed whether recent infection was associated with clustering within the inferred network.

**RESULTS:**

Recent infection was detected in 11% (22/194) of newly-diagnosed participants. Out of the participants with sequence data, 60% (9/15) with recent infection clustered compared with 31% (43/139) with chronic infection. Two recent infections belonged to the same cluster. In adjusted analyses, recent infection was associated with years of residence in Tijuana (OR = 1.5; 95%CI 1.01–1.09), cocaine use (past month) (OR = 8.50; 95%CI 1.99–28.17), and ever experiencing sexual abuse (OR = 2.85; 95%CI 1.03–7.85).

**DISCUSSION:**

A total of 11% of men newly diagnosed with HIV who have sex with men and transgender women in Tijuana were recently infected. The general lack of clustering between participants with recent infection suggests continued onward HIV transmission rather than an outbreak within a particular cluster.

## INTRODUCTION

Individuals who are in the primary stages of infection are responsible for a disproportionate number of new infections through several factors, including higher viral loads and being unaware of their status^1,2^. Rapid and targeted delivery of ART to people with recent infection may be more cost-effective at reducing transmission in resource-constrained settings than uniform delivery to similar numbers of persons living with HIV due to the increased probability of transmission during recent infection^3,4^. A study of men who have sex with men (MSM) with recent HIV infection in San Diego found that 18% of putative transmission links occurred during the putative source’s period of recency^5^. Similarly, a risk network intervention that located people with recent infection, found that the networks of those with recent infection were more likely to include others with recent infection than those with chronic HIV infections^6^.

Accurate estimates of HIV incidence among MSM and transgender women (TW) in Tijuana, Baja California, Mexico, are unavailable despite being the most heavily affected risk population in the area (HIV prevalence: MSM/TW = 20%, people who inject drugs [PWID] = 4%, female sex workers [FSW] = 6%)^7^. In the past 25 years, there have been only three estimates of HIV prevalence among MSM in Tijuana, and no reports of HIV incidence. Available estimates suggest that HIV prevalence has increased over time from 11% in a 1991 convenience sample of MSM^8^, to 18.9% in a 2002 convenience sample of MSM^9^, and ultimately 20% (95%CI 12.5–29.1) in a 2014 sample of MSM and TW recruited by respondent-driven sampling (RDS)^7^. Whereas these estimates are not sufficient to establish a trend considering the disparate and non-probabilistic sampling techniques applied, they are all notably high and have remained high over the past few decades. Reducing new transmissions is a key goal in the public health response to HIV^10^.

Preventive interventions can be directed towards the uninfected as well as the infected population, treatment as prevention (TasP) has reduced incident infections in HIV serodiscordant couples^11,12^. However, despite universal access to HIV care in Mexico, knowledge of one’s HIV status and subsequent engagement in HIV care are low^7^. The study of MSM and TW in 2014 found that out of those who tested positive, only 11% were previously aware of their HIV status.^7^ Estimates of ART use among MSM and TW are not available^7^. However, in a pooled sample of data from multiple studies assessing PWID, FSW, and MSM, the prevalence of ART use was only 3.7% overall, indicating that the rate among MSM is also quite low^13^. High rates of undiagnosed infection and subsequent low rates of linkage to care among MSM in Tijuana hinder successful treatment, leading to continued transmission. Since Mexico is a resource-limited country, it may be unrealistic to target all MSM and TW who are potentially out-of-care. Therefore, this study aims to estimate and characterize recent HIV infections among MSM and TW in Tijuana, which can serve as a starting point for effective resource allocation.

## METHODS

### Study Population

Data come from *Proyecto Enlaces* (Links Project), which was designed to compare the effectiveness of partner contact tracing (PCT) (changed to respondent-driven sampling [RDS] after 9 months) and venue-based sampling (VBS) for identifying undiagnosed infection among MSM and TW in Tijuana (as described elsewhere)^14^. PCT (eliciting and contacting sexual partners of participants who were recently diagnosed with HIV) was used between March and December of 2015^15^. RDS, a chain-referral sampling technique that modifies snowball sampling by limiting the number of individuals a participant can recruit, was used between January 2016 and November 2018. VBS was conducted between March 2015 and November 2018 at 36 venues identified via formative research as locations attended by MSM and TW to meet sexual partners (e.g., nightclubs, bars, public spaces, motels). Individuals identified via VBS were eligible for HIV testing if they were at least 18 years old, cisgender male or transgender female, reported anal sex with a cisgender male or transgender female in the past four months, and they did not report a previous HIV diagnosis.

Eligibility criteria for RDS seeds included: 1.) Persons assigned male sex at birth identifying as cisgender male or transgender female, 2.) ≥ 18 years of age, 3.) Anal sex with a cisgender male or transgender female in the past 4 months, 4.) Tijuana resident, and 5.) Knowing ≥ 15 MSM or TW who are ≥ 18 years old in Tijuana (changed to 5 MSM or TW in April 2017 to boost RDS recruitment). RDS seeds received three vouchers (changed to six vouchers to boost RDS recruitment in January 2018) to recruit MSM or TW peers from their social networks to the study. Peer-recruits were then eligible to recruit three to six of their peers if they were at least 18 years old, cisgender male or transgender female, and reported anal sex with a cisgender male or transgender female in the past 12 months. Those who did not report a previous HIV diagnosis were also eligible for HIV testing. RDS was initiated by 33 seeds who were identified by VBS or referrals from Tijuana’s municipal HIV clinic, and they were selected to be diverse regarding HIV status, age, socio-economic status, sexual orientation, gender identity, and recruitment venue.

Individuals could be identified multiple times by the same or a different recruitment method. Those identified more than once who remained eligible for HIV testing were re-tested if it had been at least three months since their last test. Eligible individuals identified via VBS underwent rapid HIV testing at recruitment venues or at the study site if they preferred, whereas those identified via RDS underwent rapid HIV testing at the study site.

Participants answered a set of questionnaires designed to collect socio-demographic, behavioral, psychosocial, and socio-contextual data, as well as detailed data on up to 20 of their sexual partners in the past four months. Each participant (regardless of recruitment method) received 150 Mexican (MXN) pesos for HIV testing and 450 MXN pesos for completing the study questionnaire (equivalent to approximately 8 and 24 US dollars), and those recruited with RDS received an additional 100 MXN pesos (approximately 5 US dollars) for each successful recruit enrolled into the study. All participants signed a written informed consent and research methods were reviewed and approved by the Institutional Review Boards at the University of California, San Diego and Xochicalco University in Tijuana, Mexico. All procedures were conducted in accordance with the 1964 Helsinki Declaration and its amendments.

### Laboratory Testing

All participants provided blood samples for serologic testing and HIV sequencing. Rapid test-positive individuals provided an additional blood sample for confirmatory testing via immunofluorescence assay at the San Diego County Public Health Laboratory and were offered enrollment in *Proyecto Enlaces*. Confirmatory HIV test results were delivered to rapid test-positive individuals within two weeks and those confirmed HIV-positive were referred for free HIV care at Tijuana’s municipal HIV clinic. HIV-1 Limiting Antigen (LAg) Avidity testing (Sedia Biosciences, Portland, OR) was performed on blood samples provided by newly-diagnosed participants, including those diagnosed as part of this study or in the past two months for PCT index participants and RDS seeds, to distinguish recent HIV-1 infections from chronic infections with an estimated cut-off of around 130 days^16^. DNA was extracted from stabilized blood using DNAGard (Biomatrica, San Diego, CA). Polymerase chain reaction (PCR) and Sanger sequencing were used to obtain HIV-1 partial pol sequences from the extracted DNA. To maximize our ability to obtain a HIV sequence from these participants, we chose partial pol sequence from bp 2726 → 3214^17^.

### Measures

The outcome of interest was recent HIV infection determined by LAg-Avidity testing. Exposures of interest are described below, and they were collected via questionnaires administered using computer-assisted personal-interviewing (CAPI) technology by local, Spanish-speaking interviewers with experience working with sexual and gender minorities and who were also trained in non-judgmental interviewing techniques to encourage an open and honest reporting on sensitive information.

### Socio-Demographic Factors

This section included age, gender identity (cisgender male or transgender female), sexual orientation (homosexual/gay, bisexual, heterosexual/straight, or questioning), highest level of education (cannot read or write, some grade school, grade school, some secondary school, secondary school, some high school, high school, some college, college or advanced degree), employment status (unemployed, part-time or full-time).

### Substance Use and Sexual Behaviors

The following characteristics were measured: Substance use measures elicited information on lifetime and past month use of illicit drugs (marijuana, heroin, inhalants, methamphetamine, ecstasy, cocaine, tranquilizers, barbiturates, amyl nitrites (poppers), γ-hydroxybutyric acid (GHB), ketamine and other). Sexual behaviors were derived from a sexual network survey, which asked about up to 20 anal or vaginal sex partners in the past four months^20^.

### Socio-Structural Factors

This section included the following: length of time living in Tijuana (years), deportation from the United States (US), homelessness (in previous four months), and incarceration history (lifetime). History of abuse was assessed via three separate questions about whether participants had ever been forced or coerced to have sex against their will, physically abused (i.e., hit or assaulted), or emotionally abused, and whether they had experienced abuse in the previous 4 months. Internalized stigma related to having sex with men was measured among MSM participants using a 9-item scale^21^. Participants indicated their level of agreement with the scale items via Likert scale responses (1 = strongly disagree, 2 = disagree, 3 = neither agree nor disagree, 4 = agree, 5 = strongly agree). We adapted the nine items presented to MSM to reflect experiences of TW (e.g., “I have tried to stop being attracted by men in general” was changed to “I have tried to stop identifying myself as a woman in general”) (Cronbach’s alpha=0.89 for adapted items), to measure internalized stigma related to gender identity among TW participants. Participants’ responses to items measuring internalized stigma were summed to create a score, with higher scores indicating greater levels of internalized stigma. Outness about having sex with men or being a transgender woman was assessed by asking participants to describe how ‘out’ they are about having sex with men or being a transgender woman on a scale of 1–7 (1 = not out to anyone; 4 = out to about half the people I know; 7 = out to everyone)^22^. Social support was measured via Likert scale responses (1 = strongly disagree, 2 = disagree, 3 = agree, 4 = strongly agree) with 7 items on help and support received from friends and family (e.g., *“*Do people close to you let you know they care about you?*”*). Social support scores were computed by averaging responses across items and transforming the result average to a score on a 100-point scale, with higher total scores indicating greater social support^23^.

### Inference of HIV Transmission Network

HIV-1 *pol* sequences were aligned using MUSCLE in AliView^24^ and analyzed to infer an HIV transmission network using HIV TRAnsmission Cluster Engine (HIV-TRACE)^25^. After aligning sequences to an HXB2 reference sequence, Tamura-Nei 93 (TN93) genetic distances were measured between all pairs of sequences. To construct the HIV transmission network, participants whose sequences had TN93 genetic distances ≤ 0.015 substitutions per site were connected by putative HIV transmission links. This genetic distance threshold has been validated to identify partners with direct or indirect epidemiological links^26^, and it is used for molecular HIV surveillance in U.S. public health^27^. It is a widely used threshold for non-outbreak related research^28^. Clustering was defined as having ≥ 1 putative transmission link within the inferred network.

### Statistical Analysis

Unadjusted and adjusted logistic regression models were used to assess the relationship between recent HIV infection and participant’s characteristics across the following domains: 1. Socio-demographics, 2. Socio-structural factors, and 3. Substance-use and sexual behaviors. Characteristics with large magnitudes of association in unadjusted models were considered as exposures of interest in separate adjusted models to estimate the total effect of each exposure of interest on recent HIV infection^33^. Potential confounding variables were selected for inclusion in adjusted models based on *a priori* knowledge about their interrelationships with the exposures of interest and recent HIV infection. All regression analyses were conducted using Stata version 15. Assortativity was estimated within the inferred HIV transmission network regarding recent infection (i.e., the tendency for participants with recent infection to have putative transmission links to other participants with recent infection) to assess whether recent infections might be the result of an outbreak or ongoing transmission^34^. To assess the statistical significance of observed assortativity patterns within the inferred transmission network considering the relative representation of the duration of HIV infection (recent vs. chronic) in these clusters, we generated expected distributions for the assortativity parameter by randomly permuting the duration of HIV infection labels on the static network 5000 times using Python 3.6.8 and SciPy. 1.3.1.

## RESULTS

In total, 232 HIV-positive MSM and TW were enrolled in *Proyecto Enlaces*. Out of which, 203 were newly diagnosed HIV-positive (188 as part of the study) and 175 had HIV-1 *pol* sequence data (sequences could not be generated from blood samples provided by 36 participants). Out of newly-diagnosed participants, 198 received the LAg Avidity testing and 155 had both sequences and LAg results. The median age of the participants with LAg test results (n = 195) was 31 (IQR 24–39). Most participants identified themselves as male (90%), were employed (62%), and were stably housed in the previous 4 months (85%). Half of the sample reported having at least high school education (50%), and just over a quarter reported a history of incarceration (28%). Most participants reported identifying as gay/homosexual (57%), while 32% identified as bisexual, 10% identified as straight/heterosexual, and 2% were questioning. Out of those who identified themselves as straight/heterosexual, 58% identified as transgender. The median length of time lived in Tijuana was 9 years (IQR 3–19). Most participants were born in Mexico (95%), with only 32% born in the state of Baja California. Thirty-one percent reported ever being deported from the US. The most commonly reported drugs used in the previous month were methamphetamine (34%) and marijuana (32%). Eleven percent reported injecting drugs in the past month, and 61% reported using drugs before sex in the past 4 months. The median number of sexual partners in the previous 4 months was 4 (IQR 1.5–9). Regarding socio-structural factors, 48% reported ever experiencing physical abuse and 39% reported ever experiencing sexual abuse. The median scores on the internalized stigma scale, outness scale, and social support scale were 21 (interquartile range [IQR] 17–30), 5 (IQR 2–7), and 66 (IQR 38–88), respectively.

Recent infection was detected in 11% (22/194) of newly-diagnosed participants. A breakdown of select characteristics by the duration of HIV infection (recent vs. chronic) can be found in Table 1. In adjusted analyses, recent infection was associated with length of time living in Tijuana (OR = 1.05 per year; 95%CI 1.01–1.09), cocaine use in the previous month (OR = 7.50; 95%CI 1.99–28.17), and ever having experienced sexual abuse (OR = 2.85; 95%CI 1.03–7.85) (Table 2). The results were not sensitive to type of recruitment.

**Table 1:** Estimated number and characteristics, and unadjusted logistic regression of factors associated with recent HIV infection among MSM/TW in the Enlaces Cohort, Tijuana, Baja California, Mexico (N = 195).

Characteristic	Incident infection n = 22 n (%)	Chronic infection n = 172 n (%)	OR	95%CI
Transgender woman	1 (6)	18 (11)	2.15	0.27–17.17
Age, years (median, IQR)	33 (23–44)	31 (26–39)	0.99	0.95–1.05
Education > high school	12 (57)	81 (49)	1.40	0.56–3.50
Unemployment	8 (38)	62 (37)	0.97	0.38–2.47
Time of residence in Tijuana, years (median, IQR)	19 (9–23)	8 (2–18)	**1.05**	**1.01–1.09**
Sexual orientation				
Gay/homosexual	11 (52)	94 (57)	-	-
Straight/heterosexual	0	19 (11)	-	-
Bisexual	8 (28)	52 (31)	-	-
Questioning	2 (9)	1 (< 1)	-	-
Ever deported from the U.S.	3 (14)	56 (34)	-	-
Homeless (past four months)	1 (6)	26 (16)	0.31	0.04–2.40
Ever incarcerated	3 (14)	51 (31)	0.38	0.11–1.34
Number of sexual partners (median, IQR)	3 (1.5–4.5)	4 (1–10)	0.98	0.94–1.02
Substance use (past month)				
Marijuana	10 (48)	51 (31)	2.05	0.82–5.13
Cocaine	5 (24)	8 (5)	**6.21**	**1.82–21.25**
Methamphetamines	4 (19)	61 (37)	0.41	0.13–1.27
Amyl nitrate (poppers)	4 (19)	17 (10)	2.08	0.63–6.89
Intravenous drugs	1 (5)	20 (12)	0.37	0.05–2.89
Ever experienced sexual abuse	11 (61)	58 (36)	**2.76**	**1.02–7.52**
Ever experienced physical abuse	6 (33)	78 (49)	0.53	0.19–1.49
Internalized stigma related to being MSM/TW (median, IQR)	25 (18–34)	20 (17–30)	1.04	0.99–1.10
Outness scale (median, IQR)	4.5 (1–7)	5 (3–7)	0.91	0.75–1.11
Social support scale (median, IQR)	75 (52–92)	59 (34–88)	1.01	0.99–1.03
Recruitment method				
PCT	3 (14)	19 (11)	1.38	0.36–5.36
RDS	7 (32)	48 (28)	1.28	0.47–3.44
VB	12 (54)	105 (61)	Ref.	
Clustered in transmission network	9 (60)	43 (31)	**3.35**	**1.12–9.99**

OR: odds ratio; 95%CI: 95% confidence interval; IQR: interquartile range; PCT: partner contact tracing; RDS: respondent driven sampling, VB: venue based.

Note: significant values are shown in bold.

**Table 2:** Adjusted Logistic Regressions: Factors Associated with Recent HIV Infection among MSM and TW among the Enlaces Cohort, Tijuana, Baja California, Mexico (N = 187).

	OR	95%CI	p
Years living in Tijuana	1.05	1.01–1.09	0.03
Cocaine use (past month)	7.50	1.99–28.17	0.003
Ever experienced sexual abuse	2.85	1.03–7.85	0.04

OR: odds ratio; 95%CI: 95% confidence interval

Note: Models adjusted for age and education

**Figure 1:**
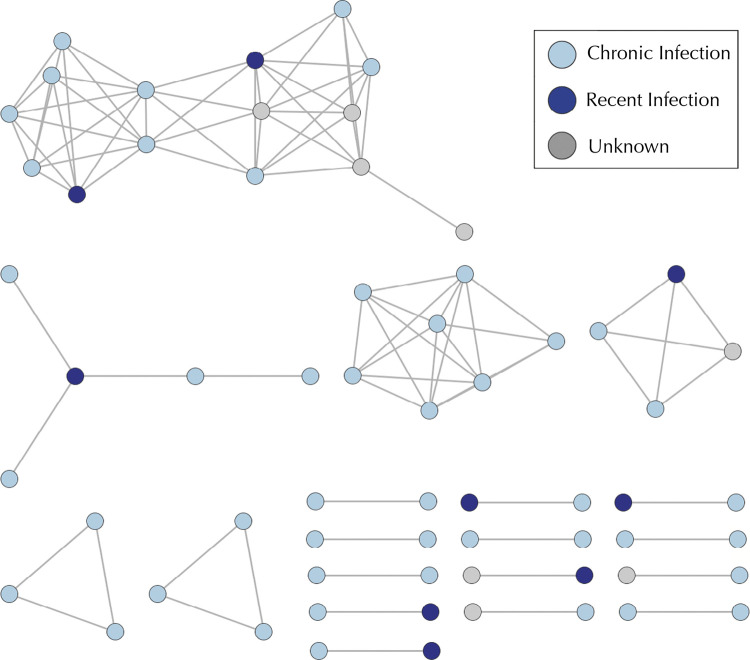
Location of recent HIV infections within the HIV transmission network.

**Figure 2:**
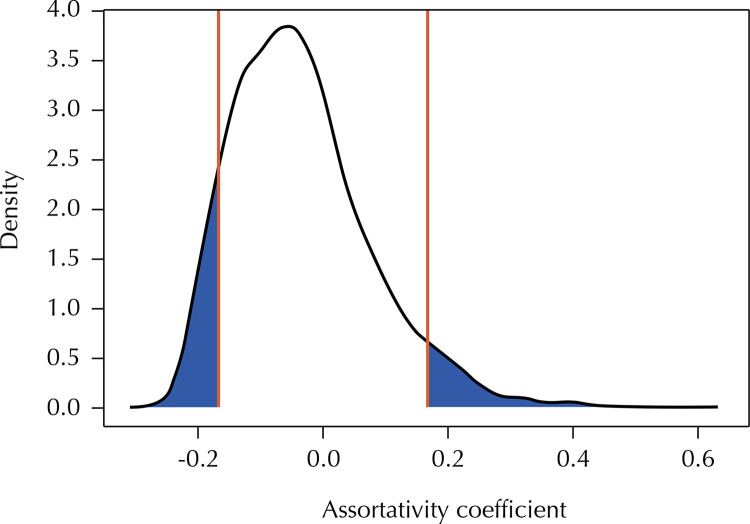
Comparison of observed assortativity coefficient and assortativity coefficients computed from 5000 randomly generated networks (reference line indicates observed assortativity coefficient).

Of the 154 newly diagnosed participants with available sequence and LAg data, 60% (9/15) of those with recent infection clustered compared with 31% (43/139) of those with chronic infection. Figure 1 represents a network of potential transmission partners: participants whose sequences had genetic distances ≤ 0.015 substitutions per site connected by putative HIV transmission links. As depicted by the figure, there were 19 clusters and the maximum cluster size was 15 persons. Two recent infections belonged to the same cluster, but the rest were in separate clusters. The assortativity coefficient was -0.168 (two-tailed, p = 0.13). Figure 2 depicts the results of the permuted distribution compared to the observed assortativity coefficient. Each permutation retained the original network structure, but randomly assigned the recent infections throughout the network. The two-tailed p-value of 0.13 reveals that the null hypothesis (recent infections do not cluster together) cannot be rejected.

## DISCUSSION

We found that 11% of MSM and TW who were newly diagnosed with HIV likely acquired it within 130 days of their HIV test (i.e. were recently infected with HIV). Recent HIV infection was associated with the length of time living in Tijuana and cocaine use in the past month. Considering the low proportion of participants who were born in Baja California (32%), this finding may indicate that MSM and TW from other areas of Mexico migrate to Tijuana for economic opportunities, and Tijuana also provides more opportunities to test for HIV^35^. As such, Tijuana may provide more access to HIV prevention and care services than more rural areas, increasing HIV testing rates and consequently our ability to detect recent infections among those who have lived in the area for longer periods.

Furthermore, we found an association between recent HIV infection and ever experiencing sexual abuse. This finding is congruent with previous literature showing that sexual abuse has been associated with increased number of sexual partners, condomless anal intercourse, condomless anal intercourse with a HIV serodiscordant partner, and it is commonly reported by Latino MSM than White MSM in the U.S.^36,37^. The mechanism behind this association is not well understood as mental health indicators such as depression and anxiety have not been found to mediate the association^38^. Interestingly, MSM and TW in Tijuana with a history of sexual abuse were also more likely to have been tested for HIV in their lifetime compared to those who have not experienced sexual abuse^38^.

The low magnitude of the estimated assortativity coefficient reveals that, in our sample, recent infections were not likely to cluster together within the inferred HIV transmission network. This probably suggests that a continued onward HIV spread occurs along multiple different transmission chains among MSM and TW population in Tijuana, rather than an outbreak within a particular cluster. These results also suggest that chronically infected individuals may be contributing to new infections in this population.

Our study is limited in its ability to infer causality between participants’ characteristics and recent HIV acquisition due to the cross-sectional nature of the study design. Moreover, the sample size was relatively small, and consequently the HIV transmission network is under sampled. Despite these limitations, this study serves as evidence of substantial ongoing transmission within the population, which calls for the need of a population-based HIV incidence estimate in the region.

## CONCLUSIONS

This study identified that 11% of newly HIV diagnosed MSM and TW in Tijuana were recently infected with HIV. The lack of clustering between the recent infections suggests continued onward HIV transmission among MSM and TW population rather than an outbreak within a particular cluster. Considering the large proportion of HIV-positive MSM and TW in Tijuana who are unaware of their infection, our results suggest a broad, active, and relatively undefined HIV epidemic among these populations in Tijuana. Considering the resource-constrained context, results indicate that resources should be allocated to those with characteristics associated with recent infection (longer duration in Tijuana, current cocaine use, and history of sexual abuse) as they are most likely to be recently infected. This study also highlights the need to obtain a high-quality estimate of HIV incidence across Tijuana to monitor the epidemic and track the impact of interventions.
